# The Human Breast Microbiome: From Homeostasis to Malignancy, Mechanistic Insights and Therapeutic Perspectives

**DOI:** 10.3390/ijms27114723

**Published:** 2026-05-24

**Authors:** Mysoon M. Al-Ansari, Suha M. Mahmood, Monther Al-Alwan

**Affiliations:** 1 Department of Botany and Microbiology, College of Science, King Saud University, Riyadh 11451, Saudi Arabia; 443204671@student.ksu.edu.sa; 2 Innovation and Research, King Faisal Specialist Hospital and Research Centre, Riyadh 11211, Saudi Arabia; 3 College of Medicine, Al-Faisal University, Riyadh 11533, Saudi Arabia

**Keywords:** breast cancer, microbiome, metagenomics, therapy resistance, dysbiosis

## Abstract

Although human mammary glands were traditionally considered sterile, accumulating evidence has established the presence of distinct microbial communities that may have colonized breast tissue primarily via retrograde nipple flow or via hematogenous or lymphatic translocation from other body sites. Comparative studies reveal differences in the microbiota of healthy and diseased breast tissues, with variations in microbial signatures across breast cancer subtypes and in comparison with adjacent normal tissues. This review synthesizes current evidence on the composition of the breast microbiome, the factors shaping its development, and alterations it undergoes in inflammatory and malignant breast diseases. Furthermore, the article discusses mechanistic insights, methodological challenges, and future therapeutic perspectives based on published studies employing culture-independent approaches, such as 16S rRNA gene sequencing and metagenomic analyses. Key host-related factors influencing breast-associated microbial communities, including hormonal regulation, environmental exposure, diet, and therapeutic interventions, are explored. The existing literature is assessed to identify key associations between the breast microbiome and host signaling pathways, as well as the significant challenges that remain unresolved, including low biomass contamination, inter-study variability, limited longitudinal data, and an incomplete understanding of causality. Addressing these limitations is critical for advancing microbiome-based diagnostic and therapeutic strategies for breast disease.

## 1. Introduction

### 1.1. The Human Microbiome: A Brief Overview

**Figure 1:**
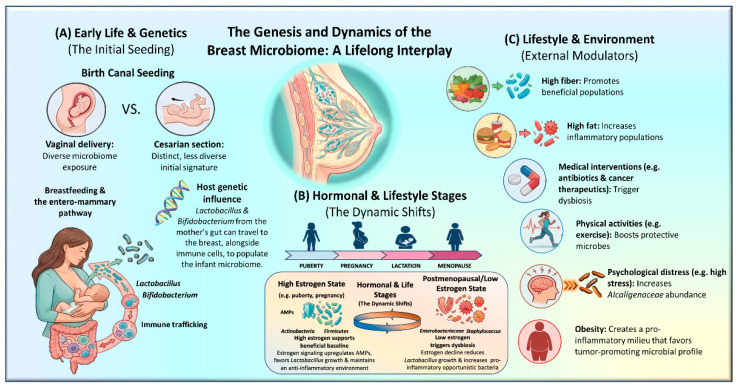
Schematic summarizing the dynamic seeding and lifelong modulation of the human breast microbiome. The central illustration of the mammary gland is surrounded by three primary categories of influencing variables: (**A**) Early Life and Genetics, highlighting initial colonization via the birth canal and maternal–infant seeding through the entero-mammary pathway; (**B**) Hormonal and Life Stages, depicting shifts across puberty, pregnancy, lactation, and menopause. Notably, high estrogen levels support beneficial lactic acid bacteria (e.g., *Lactobacillus*) and antimicrobial peptide (AMP) expression, whereas postmenopausal estrogen decline is associated with dysbiosis; and (**C**) Lifestyle and Environment, illustrating the impact of external determinants, such as dietary patterns (high-fiber vs. high-fat), antibiotic exposure, physical activity, psychological stress, and metabolic syndrome/obesity on the microbial composition. Collectively, these factors dictate the balance between local tissue homeostasis and disease susceptibility.

The microbiome encompasses microbial communities (viruses, bacteria, fungi, and archaea) and their collective genomes that naturally coexist in a given habitat [1]. Recent developments in shotgun metagenomics, an untargeted sequencing method that analyzes the entire DNA content of a sample to determine species-level taxonomic profiles and functional genes without PCR amplification of specific targets, have facilitated the precise characterization of low-biomass breast tissue microbiota. This approach helps distinguish authentic tissue-resident microbes from potential contaminants [2]. The microbiome is essential for vitamin biosynthesis, immune system maturation, xenobiotic metabolism, and maintenance of epithelial barrier integrity [3,4]. Recent multiomics studies have revealed that host genetics account for only a minor fraction of microbiome variation, with environmental exposure and diet exerting a stronger influence [5]. Multiple factors—such as heredity, environment, and nutrition—influence the diversity of the human microbiome, creating a unique fingerprint for each individual (Figure 1) [6]. Any alteration to the microbiome is a double-edged sword, as it may act as a protective barrier that benefits human health or contribute to the emergence of disease conditions, including inflammatory bowel disease [7]. Our understanding of microorganisms in the human microbiome has expanded with advances in sequencing and in the study of bacterial metabolic activity using metagenomics and metabolomics [8]. Focused investigations into the intricate interactions between humans and their indigenous microbiota may yield valuable insights into how microbiome alterations contribute to disease development and could ultimately support the identification of novel therapeutic strategies for microbiome-associated disorders.

For clarity and consistency throughout this review, the following operational definitions are used: “Commensal” refers to microorganisms that are normally present within the host and do not cause harm, although they are not necessarily beneficial. “Probiotic” refers strictly to live microorganisms that provide a demonstrated health benefit when administered. “Beneficial” describes taxa associated with positive health outcomes in correlative studies. Finally, “dysbiosis” refers to a pathological shift in the microbiome, which may encompass a loss of diversity, depletion of potentially beneficial taxa, or enrichment of pathobionts.

### 1.2. Distinctive Features of the Breast Microbiota

The breast tissue harbors a distinct, low-biomass microbiome that differs from those of the skin, gut, and oral cavity [9,10]. While the breast microbiome exhibits significant inter-individual variability, it is generally characterized by a lower alpha diversity, indicating lower species richness and evenness within tissue samples. Several studies consistently identify genera such as *Staphylococcus* and *Streptococcus* as common constituents of the breast microbiota, which are considered core commensal organisms [9,11]. Under normal physiological conditions, these bacteria may contribute to immune regulation and tissue homeostasis. However, disruptions in microbial balance (dysbiosis) have been associated with inflammatory processes, tumor progression, and metastasis, underscoring the potential role of the breast microbiome in both health and disease [12,13]. The breast tissue, milk, and milk ducts harbor a wide variety of normally living microorganisms, collectively known as the microbiome, given their genomic and metabolic systems [14]. This microbiota not only affects breastfeeding outcomes but also contributes crucially to maintaining breast health. The dynamic nature of the breast microbiome, influenced by factors such as hormonal fluctuations, nursing practices, and the mother’s health, is one of its unique characteristics [9]. Moreover, dysbiosis in the breast microbiome could influence the health of a newborn through breastfeeding and possibly cause diseases, such as mastitis [15,16]. Recent mapping studies have shown that microbial communities in tumor-adjacent normal tissue differ from those in tumor cores, suggesting distinct microenvironmental niches [17]. Establishing the link between breast microorganisms in mothers and newborn health requires a deep understanding of breast microbiome composition and function. Moossavi et al. [18] suggested that in-depth investigations into the dynamics of the breast microbiome could yield innovative approaches for enhancing maternal well-being and optimizing newborn nutrition. A study further suggested that mother–child microbiological transmission during breastfeeding may influence the development of the offspring’s immune system and metabolic functions [19].

## 2. Origin of the Breast Microbiome

### 2.1. Sources of Initial Colonization

Environmental factors, including geographic location, significantly shape breast microbial composition [20]. Throughout life, the breast and milk microbiome is a dynamic, complex community influenced by multiple factors, including hormonal fluctuations, antibiotic exposure, and environmental exposure [21,22]. Aside from being crucial for the general health of the mammary glands, the presence of certain pathogenic bacteria in the breast may also affect a person’s vulnerability to conditions such as mastitis or even breast cancer. Developing measures to support microbial diversity and balance within this niche requires an understanding of the origins of initial colonization and the variables influencing the composition of the breast microbiome [15,16].

### 2.2. Maternal–Infant Microbial Seeding via the Birth Canal and Breastfeeding

Breastfeeding and passage through the birth canal are important processes that seed an infant’s microbiome and influence its diversity and composition. During vaginal delivery, infants are directly exposed to maternal vaginal and perianal microorganisms, enabling a wide range of bacteria to colonize the gut [23]. Dominguez-Bello et al. [24] demonstrated that this early microbial exposure facilitates the development of a healthy infant microbiome and influences metabolic activities. Furthermore, breastfeeding supplies live beneficial bacteria, such as *Lactobacillus* and *Bifidobacterium*, as well as human milk oligosaccharides that serve as prebiotics, promoting the colonization and growth of healthy gut microbes in infants [25,26]. The special nutritional composition of breast milk supports immune development in infants and maintains their gut health by preventing infections and inflammatory diseases [27,28]. A comprehensive meta-analysis of human milk microbiome studies revealed the consistent detection of core bacterial genera, such as *Streptococcus*, *Staphylococcus*, *Corynebacterium*, and *Lactobacillus*, across diverse populations, with significant inter-individual variation depending on lactation stage, delivery mode, and maternal factors [18,29]. Metagenomic studies have demonstrated that the breast microbiome, which is not derived solely from the skin or the infant’s oral cavity, also harbors unique taxa (e.g., *Methylobacterium*, *Ralstonia*, and *Sphingomonas*) [10,22]. These microbes may be transferred via the entero-mammary pathway, in which maternal gut bacteria or bacterial components reach the mammary gland via immune cell trafficking or lymphatic/hematogenous routes, ultimately seeding the infant gut microbiota [29]. Furthermore, nursing alters the microbiological composition of breast tissue, which may affect maternal health-related outcomes, including mastitis [30,31]. Collectively, the evidence supports bidirectional interactions between the maternal breast microbiota and infant colonization; however, determining the causal mechanisms and long-term health impacts requires longitudinal, well-controlled studies.

## 3. Evolution and Development of the Breast Microbiota

### Genetic and Early-Life Determinants of Microbiome Establishment

The breast microbial population is dynamically regulated by genetic, hormonal, and environmental factors (Figure 1). Host genetics is a major variable influencing the development of the breast microbiome. Mechanistically, an individual’s genetic makeup can modulate epithelial receptor expression and immune responses, thereby determining which microbes thrive and colonize the breast tissue [9,32]. Genetic polymorphisms can influence epithelial surface receptors, mucosal secretions (e.g., glycans), and local immune responses through pattern recognition receptors, all of which are crucial in controlling microbial adherence and proliferation in the gut. Variations in genes related to mucin glycosylation or immune signaling may favor or inhibit colonization by specific bacterial taxa. This model is consistent and supports multiple reports demonstrating that host genetics broadly influence microbiome composition [5] and is parallel with studies suggesting genetic regulation of the breast duct microbiota [9]. Ethnic and geographic differences in the breast microbiome composition may contribute to disparities in breast cancer incidence and outcomes [33]. The delivery procedure during childbirth is another important factor affecting the breast microbiota. Compared with babies born via vaginal delivery, those born via cesarean section exhibit distinct patterns of microbial colonization in the stomach and breast tissue [24,34]. This variability in microbial communities could have severe consequences for immunological development and the general health of infants [24]. During lactation, the total bacterial counts, especially those of *Enterococcus* spp., and *Bifidobacterium*, increase. Various studies demonstrate that *Bifidobacterium* spp. are enriched in infants delivered vaginally. Differences in colostrum composition have also been noted between delivery modes [24,35].

Furthermore, the breast microbiota composition in women is dynamically affected by hormonal changes during major life stages, including puberty, menstruation, pregnancy, lactation, and menopause. Thus, breast microbial diversity and abundance are broadly affected during these milestones [10,36]. For example, the total microbial population varies throughout pregnancy to support fetal development, then changes again during lactation to aid breast milk production. Later in life, the cessation of the menstrual cycle during menopause introduces further shifts in the breast microbiome. The specific mechanisms by which hormonal fluctuations, particularly those of estrogen, modulate the breast microbiome will be discussed in detail in Section 9.

## 4. Composition of the Breast Microbiome

### 4.1. Key Bacterial Taxa and Core Microbiome Signatures

Developments in sequencing technology have helped identify important bacterial species found in breast tissue [37]. Urbaniak et al. [9] showed that breast milk harbors several probiotic bacteria, including *Streptococcus*, *Lacticaseibacillus*, *Staphylococcus*, *Leuconostoc mesenteroides*, and *Lacticaseibacillus rhamnosus*. Moreover, research has demonstrated that variables, including age, hormonal state, and lactation, can affect the composition of the breast microbiome. While healthy breast tissue has a more diverse microbiome, breast cancer tissues are more abundant in bacteria linked to inflammation, such as *Enterobacteriaceae*, *Staphylococcus*, and *Escherichia coli* [10,16,38,39]. This underscores the significance of understanding the bacterial composition of the breast, where dysbiosis in the microbiome may be associated with the development of breast cancer [22,40].

Distinct microbial taxa vary in abundance between healthy and malignant breast tissues (Table 1). This finding suggests that specific bacterial strains may be associated with various breast pathologies, highlighting the need for further investigations into microbial communities across diverse medical circumstances. The environment of the breast microbial population is dynamic and complex, and is influenced by numerous factors. Further studies are required to identify the pathways by which the breast microbiome influences breast health and disease [41].

### 4.2. Comparative Microbiome Profiles: Healthy vs. Malignant Breast Tissues

The breast microbiome is pivotal in preserving breast health. The microbial composition varies significantly between healthy individuals and those with breast conditions. Xuan et al. [10] discovered that tissue samples from patients with breast cancer had substantially less microbial diversity than those from healthy controls, indicating a possible link between changes in the breast microbiome and the emergence of breast cancer. Furthermore, the bacterial species present differ between patients with breast cancer and healthy individuals. Women with benign breast disease exhibit greater numbers of certain bacteria, including *Streptococcus* and *Staphylococcus,* than healthy individuals [39]. *Escherichia coli* and *Proteobacteria* spp. are enriched in certain tumor cohorts, with variations observed across different molecular subtypes of breast cancer [38,42]. In addition to these bacteria, relatively low abundance of Firmicutes, Actinobacteria, and Bacteroidetes have been identified [43]. Thus, a significant variation in specific bacterial species in the cancer microbiome, or dysbiosis, may promote the emergence or progression of breast diseases.

Microbial diversity, species composition, and functional properties vary between diseased and healthy individuals. Costantini et al. [44] found altered metabolic pathways in patients with breast cancer, including modifications in carbohydrate metabolism and amino acid production. Bacteria-induced DNA damage alone is insufficient to induce cancer, but it can become influential when combined with other factors [45,46]. Elevated levels of 5-alpha-3,20-dione generated from progesterone have been observed in tumor tissues compared with adjacent normal tissues. Accordingly, *Bacillus cereus* shows similar metabolic activity, and increased levels of 5-alpha-3,20-dione have been reported in cancer cells compared with healthy cells [46]. These findings imply that resident microbiome-mediated metabolic processes contribute to the pathophysiology of breast diseases. They also emphasize the importance of breast bacterial flora in maintaining health and how alterations in its composition or function could influence the development of breast disorders. Novel therapeutic and diagnostic approaches for breast disorders must be developed based on a deep understanding of the variations in bacterial populations between healthy and diseased states.

### 4.3. Microbial Diversity as a Determinant of Mammary Health

The general health of the human body, including breast tissue health, depends heavily on microbial diversity. In particular, the wide range of bacteria, fungi, and viruses that comprise the breast microbiota exhibit functional properties in the mammary gland [47]. According to several studies, the total microbial population variance in the breast can significantly impact breast health and may contribute to cancer progression and other illnesses. Understanding the distribution and function of bacteria in the breast is essential for developing novel preventative and therapeutic approaches for breast-related diseases [48]. Specific microbial species (e.g., *Fusobacterium nucleatum*) can induce the production of immune factors and pro-inflammatory cytokines in the breast, thereby exacerbating the risk of breast cancer and other inflammatory disorders [49,50]. In contrast, certain beneficial bacteria, such as *Lactobacillus* and *Bifidobacterium*, exert anti-inflammatory properties that support the normal balance of breast immunological responses [44,51]. Urbaniak et al. [9] showed that regulating the breast microbiota can effectively prevent or treat breast cancer and other breast diseases. Preventing harmful bacterial growth, which can induce infections and other health issues, is one of the fundamental roles of the breast microbiota. Beneficial bacteria can reduce infection risk by preventing harmful pathogens from accessing nutrients and tissue space within the breast. The potential anticancer effects of the breast microbiota are highlighted by studies demonstrating how specific microorganisms (e.g., *Bifidobacterium* spp.) may inhibit the proliferation of breast cancer cells [44,52]. Further research is essential to elucidate bacterial diversity and explore the therapeutic potential of modifying the breast microbiome [53]. Certain bacteria have been identified that can alter immune function, creating a tumor-permissive environment [54]. The bacterial counts of Staphylococcus spp., which are involved in inflammatory responses, are elevated in untreated tumors [55], suggesting that cancer treatments may be influenced by the types of microbial populations in the breast microbiome and by the anti-inflammatory responses of beneficial microbes. The composition of the available microbiota may affect the efficacy of chemotherapy and other breast cancer therapies, thereby influencing patient outcomes [56,57]. Therefore, altering the breast microbiome through probiotics or other treatments may be a novel way of boosting the efficacy of breast cancer treatments [52]. Risk factors, such as aging, diet, family and personal history, obesity, and environmental factors, also influence breast cancer. Breast and gut microbial population changes are severely associated with breast cancer [58]. Future studies examining the potential relationship between microbiome availability and breast health may yield novel strategies to develop preventive measures to manage breast illnesses [59].

## 5. Interactions Between Host Cells and Microbiota

### Mechanisms of Host-Microbiota Crosstalk in the Breast

**Figure 2:**
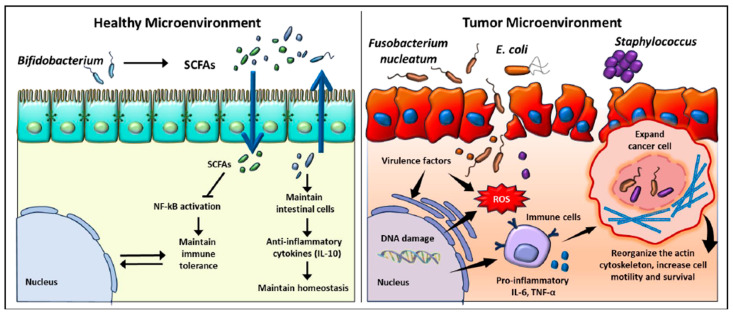
Comparative representation of the breast tissue microenvironment transitioning from microbial homeostasis to malignancy. (**Left**) Under normal physiological conditions, an intact epithelial barrier is maintained by commensal bacteria (e.g., *Bifidobacterium*). These beneficial microbes produce short-chain fatty acids that confer anti-inflammatory protection by suppressing pro-inflammatory cytokines and preventing NF-κB activation. (**Right**) Dysbiosis induces the overgrowth of pathogenic taxa, triggering chronic inflammation, immune modulation, and the breakdown of epithelial barrier integrity. Furthermore, the malignant microenvironment is characterized by microbial-induced DNA damage and the presence of intracellular tumor-resident bacteria. Intracellular microbes localize directly within the cytoplasm of cancer cells, where they reorganize the host actin cytoskeleton to enhance cellular motility, thereby promoting cell survival during metastatic dissemination.

The mammary gland microbiota influences breast epithelial cell proliferation and immune homeostasis through a complex network of signaling molecules [60,61]. Beneficial commensal strains produce short-chain fatty acids (SCFAs), which exert anti-inflammatory effects that inhibit NF-kB activation and regulate epithelial cell proliferation [45]. Conversely, dysbiosis is linked to chronic inflammation. Specific bacteria can trigger the production of pro-inflammatory cytokines. For example, *F. nucleatum* utilizes virulence factors like lipopolysaccharide and FadA, a unique filament-forming adhesion protein, to upregulate matrix metalloproteinase-9 and promote cancer cell proliferation [50]. The local microbiota also maintains the epithelial barrier by regulating tight junctions and synthesizing antimicrobial peptides [44]. Interaction with the host immune system occurs via pattern recognition receptors (e.g., Toll-like receptors [TLRs]) that engage microbial lipopolysaccharides and peptidoglycans, thereby initiating cascades that regulate cytokine production, induce T-cell tolerance, and shape the gut-associated lymphoid tissue response [62]. Disruption of this bidirectional microbial–immune system crosstalk can lead to aberrant immune responses, barrier dysfunction, and a pro-tumorigenic microenvironment. Figure 2 summarizes the proposed mechanistic pathways through which the breast microbiome may contribute to carcinogenesis, including microbial-induced DNA damage, immune modulation, metabolic reprogramming, chronic inflammation, and alterations in epithelial barrier integrity.

## 6. Environmental, Metabolic, and Lifestyle Drivers of Breast Microbiome Dysbiosis

### 6.1. Diet, Environment, Toxins, and Antibiotic Exposure

Environmental factors, including diet, toxins, and iatrogenic exposures, significantly affect the composition and functions of the breast microbiome. High-fat and high-sugar diets are recognized drivers of dysbiosis, whereas a fiber-rich diet that includes fruits and vegetables supports a diverse gut microbiome. Consuming fermented foods could hypothetically increase populations of beneficial bacteria in the breast and help modulate inflammatory responses. Notably, evidence demonstrating the impact of dietary modification on the breast microbiota in humans remains very limited. Beyond diet, antibiotic use can acutely disrupt breast microbial flora. Antibiotic-induced depletion of beneficial microbial populations may stimulate local dysbiosis and influence breast-related health outcomes. Furthermore, environmental exposure to toxic chemicals, including pesticides, pollution, and endocrine-disrupting substances, can adversely affect the breast microbiome composition and function, thereby promoting pro-inflammatory states. Heavy metal exposure has also been implicated in alterations in microbial populations, with evidence suggesting potential links to mammary carcinogenesis via interactions with the gut microbiota–breast cancer axis [63].

### 6.2. Obesity and Metabolic Syndrome

Obesity and metabolic syndrome, characterized by dyslipidemia, hypertension, and insulin resistance, are fundamentally linked to chronic, low-grade systemic inflammation, which can alter microbial diversity. Hyperglycemia and insulin resistance may influence microbiota diversity by favoring the proliferation of opportunistic pathogens while decreasing the abundance of beneficial taxa. The resulting dysbiosis generates a pro-inflammatory microenvironment that promotes the survival and proliferation of breast cancer cells [64].

Distinct microbial enrichments have been observed in patients with breast cancer, including in *Paenibacillus* spp., which have been strongly associated with lower survival [65]. Furthermore, microbial-derived metabolites such as lipopolysaccharides, cadaverine, and trimethylamine N-oxide have been implicated in tumor progression. In contrast, SCFAs produced by commensal bacteria can modulate histone acetylation in breast epithelial cells, promoting the expression of tumor suppressor genes [66]. Notably, most of the current mechanistic evidence linking metabolic syndrome to microbial dysbiosis is extrapolated from gut microbiome studies; direct human evidence demonstrating the effects of dietary or metabolic modulation on breast tissue microbiota remains limited.

### 6.3. Lifestyle Determinants: Smoking, Alcohol, Physical Activity, and Stress

Lifestyle choices inherently impact systemic and local microbial populations. Smoking is a known driver of dysbiosis, with studies demonstrating that the breast tissue in smokers harbors a significantly different microbial composition from that in non-smokers [9]. Similarly, prolonged alcohol consumption has been associated with disruption of microbial equilibrium and alterations in breast microbiota diversity [67]. In contrast, regular exercise has been associated with beneficial effects on microbial diversity and the abundance of gut probiotic bacteria [68]. However, no studies have directly evaluated the specific impact of exercise on the human breast microbiome, highlighting a significant research gap. Determining the role of lifestyle factors facilitates understanding of their impact on microbial populations in breast tissue (Figure 1). Psychological stress may also affect the microbiome via the gut–brain axis. In a study evaluating the quality of life and distress levels in patients diagnosed with breast cancer using the FACT-B questionnaire and the Distress Thermometer, higher psychological distress was linked to elevated abundances of *Alcaligenaceae* and *Sutterella*. Conversely, lower psychological distress was correlated with lower *Streptococcaceae* and *Streptococcus* abundances. Consequently, managing chronic stress may support host-microbial homeostasis and regulate pro-inflammatory cytokine expression [69].

### 6.4. Iatrogenic Alterations: Surgery, Radiation, and Chemotherapy

Clinical interventions for breast cancer can actively remodel the local and systemic microbiome. Surgical procedures, such as phased post-mastectomy breast reconstruction, frequently involve antibiotic irrigation. Compared with saline treatment, prophylactic triple-antibiotic therapy significantly affects the periprosthetic capsule microbiota in cancer-naive breasts, demonstrating an iatrogenic alteration of the local microbiome [70].

Radiation therapy, which induces cancer cell death via DNA damage, also induces gastrointestinal distress and systemic dysbiosis. Radiation-induced reduction in gut microbiome diversity is associated with an increase in pathogenic *Fusobacterium* spp. and a gradual decrease in beneficial Firmicutes. Conversely, the baseline presence of robust probiotic microbial flora significantly enhances the effectiveness of radiation therapy [57]. Similarly, systemic chemotherapy critically affects the rapidly dividing cells of the gut mucosa, inducing dysbiosis that can, in turn, alter drug efficacy. In animal models, antibiotic-induced depletion of the gut flora reduced the effectiveness of cisplatin and oxaliplatin chemotherapy. Drug metabolism and host responses are actively influenced by specific microorganisms, such as *Paraprevotella clara* and *Bacteroides fragilis*, indicating that both the gut and tumor microbiota are key modulators of chemotherapeutic efficacy and drug resistance [57].

## 7. Dysbiosis and Breast Pathology

### Microbiome Signatures Associated with Breast Carcinogenesis

Breast-tissue-resident bacteria have been consistently linked with breast cancer progression [39]. However, researchers disagree on the bacteria’s origins. A recent study revealed the presence of bacteria from the family Bacteroidaceae in the mouth, tumor tissue, and gut of dogs [71], suggesting that bacteria may have been translocated from the mouth to the gut and then to the mammary gland. However, the isolation of *Staphylococcus epidermidis* and *Micrococcus luteus* from breast tumors suggests the possible translocation of these bacterial strains through the nipples and into the mammary ducts. Methodological advances over the last 5 years have significantly advanced the analytical approaches used in the field, from basic 16S amplicon profiling to high-resolution spatial mapping and metatranscriptomic analyses. For example, breakthrough single-cell RNA sequencing and spatial mapping techniques (such as INVADEseq), as reported by Fu et al. [12], explicitly demonstrated that tumor-resident bacteria are frequently intracellular, localizing within the cytoplasm of malignant cells and tumor-associated macrophages. This intracellular niche protects the bacteria from host immune surveillance. Studies using preclinical murine models have demonstrated that, upon invasion, intracellular microbes can recognize and reorganize the host actin cytoskeleton, enhancing cellular motility and resistance to mechanical stress in the circulation, suggesting a potential mechanism for promoting cancer cell survival during metastatic dissemination (Figure 2) [72,73]. However, crucially, human studies characterizing these intertumoral bacteria are mostly cross-sectional and observational. While these microbial signatures are strongly correlated with malignancy, causality in human breast cancer is not yet proven. An outstanding question remains whether these bacteria actively drive tumorigenesis and metastasis in humans, or whether the immunosuppressed and hypoxic tumor microenvironment merely provides a favorable physiological niche for opportunistic bacterial colonization from adjacent tissues or hematogenous spread. A summary of the microbial taxa identified in healthy versus cancerous breast tissue is presented in Table 1.

**Table 1:** Microbial taxa identified in healthy vs. cancerous breast tissue.

Category	Healthy Breast Tissue/Breast Milk	Cancerous Breast Tissue	References
Dominant phyla	Firmicutes, Actinobacteria	Proteobacteria, Actinobacteria	[4,9,10,11,13]
Common genera	*Staphylococcus*, *Streptococcus*, *Lactobacillus*, *Corynebacterium*, *Cutibacterium*	*Escherichia*, *Pseudomonas*, *Ralstonia*, *Acinetobacter*, *Methylobacterium*	[38,42,58]
Probiotic/beneficial species	*Lacticaseibacillus rhamnosus*, *Lactobacillus fermentum*, *Leuconostoc* spp.	Reduced relative abundance compared with healthy controls	[74]
Inflammation-associated taxa	Low abundance of opportunistic pathogens	Higher levels of *Escherichia coli*, *Streptococcus agalactiae*, and other opportunistic bacteria	[12,22,39,67]
Microbial functions	Immune regulation, maintenance of epithelial integrity, production of anti-inflammatory metabolites	DNA damage, chronic inflammation, altered estrogen metabolism, and immune evasion	[11,12,42,58]
Alpha diversity	Higher microbial diversity	Lower microbial diversity in the tumor microenvironment	[10,11,17]

Numerous studies have identified various microbial signatures associated with different subtypes of breast cancer, with variations in microbiota composition observed among the major molecular subtypes. Banerjee et al. [42] identified a link between distinct microbial signatures and specific cancer subtypes, including triple-negative breast cancer (TNBC), estrogen receptor-positive (ER+), HER2/neu-positive, and triple-positive. Smith et al. [75] reported distinct abundances of the Firmicutes, Eucaryarchaeota, and Cyanobacteria in TNBC. Furthermore, analysis of a dataset containing 668 breast tumor samples revealed the link between the expression of certain tumor genes and the microbiome profile [38]. These results indicate an association between distinct tumor-associated microbiota and intrinsic tumor traits; however, more work is needed to explore the link between the tumor-associated microbiome and mutations in breast cancer cells. Notably, *Staphylococcus* and *E. coli* isolated from breast cancer specimens exhibit distinct genotoxic activity [76]. More recently, AlDawsari et al. [77] demonstrated that factors secreted by MDA-MB-231 TNBC cells alter the metabolic profile of *Pseudomonas aeruginosa*. Similarly, emerging in vitro evidence from luminal A models indicates that tumor-derived conditioned media can reprogram breast-associated bacteria by modulating biofilm formation, antimicrobial susceptibility, and virulence gene expression in a species-specific manner [78]. These findings further support the concept that breast cancer cells actively shape the functional phenotype of the local microbiota within the tumor microenvironment. These cancer cell-derived factors not only modulate bacterial metabolism but also drive the production of specific microbial metabolites with divergent effects on tumor biology: some promote oncogenesis, while others exhibit tumor-suppressive properties. This finding underscores the complexity of the breast tissue microenvironment, where cancer cells and resident microbiota engage in dynamic crosstalk. Elucidating these complex interactions could lead to novel therapeutic interventions targeting both tumor cells and their microbial counterparts to disrupt microbiota-induced cancer progression. However, further studies are needed to clarify the underlying mechanisms and determine how these host–microbe interactions actively shape the tumor microenvironment, with potential for developing novel strategies for breast cancer prevention and treatment.

## 8. Microbial Modulation of the Local Immune Microenvironment

### 8.1. Chronic Inflammation and the Dysbiosis Feedback Loop

Observational studies have frequently correlated microbiome dysbiosis with long-term systemic inflammation and a higher breast cancer incidence [79]. However, the directionality of this relationship remains controversial. Hypothetically, dysbiotic microbiota may produce metabolites that disrupt immunological homeostasis and promote pro-tumorigenic inflammatory reactions. An equally plausible explanation is that the developing tumor, along with its highly inflammatory microenvironment, and subsequent iatrogenic treatments induce local microbial dysbiosis as a secondary consequence [80]. Zitvogel et al. [81] noted that persistent inflammation may further alter the microbiome composition, potentially generating a feedback loop that maintains a pro-tumorigenic milieu.

Pro-inflammatory cytokines, such as TNF-α, IL-1β, and IL-6, alter the local tissue environment, promoting the growth of opportunistic pathogens while suppressing beneficial commensal bacteria [82]. The resulting inflammatory state also increases reactive oxygen species levels, selecting for microbes resistant to oxidative stress. In turn, specific microbial taxa can exacerbate this state. For example, *Parvimonas micra* can activate the RAS/ERK/c-FOS pathway, thereby initiating downstream inflammatory responses that assist in tumor formation. Ultimately, this bidirectional regulation may also create a feedback loop that maintains a pro-tumorigenic milieu [83].

### 8.2. Commensal Defense Mechanisms and SCFA-Mediated Homeostasis

A balanced breast microbiome may confer protection against opportunistic infections and mitigate chronic inflammation. Beneficial commensal bacteria species, such as *Lactobacillus* and *Staphylococcus*, restrict pathogen colonization by reducing competition, lowering local pH, and secreting antimicrobial bacteriocins. Beyond direct pathogen exclusion, these microbes may modulate the local immune microenvironment by producing SCFAs. SCFAs are produced by bacterial fermentation of dietary carbohydrates and act as critical anti-inflammatory signaling molecules. They can directly inhibit nuclear factor kappa B (NF-κB) activation, thereby suppressing the biosynthesis of pro-inflammatory cytokines, such as TNF-α and IL-6 [84].

Concurrently, SCFAs and microbial interactions with host TLRs can stimulate the production of anti-inflammatory cytokines, most notably IL-10. This combined suppression of inflammatory signaling cascades and upregulation of IL-10 fosters immunological tolerance and promotes tissue repair. Collectively, these mechanisms establish an anti-tumorigenic microenvironment that resists chronic inflammation, which is a primary risk factor for breast carcinogenesis [84].

## 9. Endocrine–Microbiome Interactions

### Bidirectional Hormone–Microbiome Signaling

Hormones play a major role in shaping the resident microbiome, while the microbiota reciprocally modulates hormone bioavailability through the “estrobolome,” a set of microbial genes encoding enzymes involved in estrogen metabolism. Specifically, certain bacterial taxa, such as *Clostridia*, produce β-glucuronidase and β-glucosidase, which deconjugate excreted estrogens, facilitating their reabsorption into the circulation, thereby increasing local bioactive estrogen levels [72]. This bidirectional relationship may have important implications for hormone-dependent malignancies. Higher baseline estrogen levels have been associated with shifts in microbial composition, supporting the proliferation of beneficial bacteria (e.g., *Lactobacillus* and *Bifidobacterium*) and modulating host antimicrobial peptides, such as β-defensins [85,86]. Menopause is associated with a steep decline in estrogen levels, which may reduce antimicrobial peptide expression and contribute to microbiome dysbiosis. This menopause-induced shift has been correlated with decreased abundance of lactic acid-producing bacteria and overgrowth of opportunistic pathogens, such as Enterobacteriaceae and *Staphylococcus* [34]. Key human cohort studies involving postmenopausal women have demonstrated that increased abundance of gut *Clostridia* and elevated microbial β-glucuronidase activity are positively associated with elevated estrone levels and urinary estrogen metabolites, both of which are established risk factors for breast cancer [72]. Furthermore, dietary phytoestrogens, such as soy isoflavones and lignans, exhibit bidirectional interactions with the microbiota. They can be metabolized by the microbiota into bioactive compounds that competitively bind estrogen receptors, while fermented soy beverages produced using probiotics have demonstrated potential protective effects against breast carcinogenesis [72]. Furthermore, although hormone replacement therapy is used to manage menopausal symptoms, its ability to restore microbial homeostasis via systemic hormone modulation requires further prospective validation.

## 10. Microbiome-Targeted Therapeutics and Treatment Modulation

### Microbial Interventions and Treatment Efficacy

The use of targeted microbial interventions, including prebiotics, probiotics, and synbiotics, is a promising, albeit preliminary, theoretical approach for modulating the breast microbiome. Current evidence suggests that certain beneficial bacterial strains may modulate immune function and attenuate chronic inflammatory responses associated with breast carcinogenesis. For example, oral administration of *Lactobacillus reuteri* can inhibit breast cancer progression in controlled animal models [39]. However, extrapolating these interventional successes to humans remains highly speculative due to the lack of prospective, longitudinal clinical studies validating the efficacy of probiotics-based interventions, specifically within human breast tissue. Consequently, although microbiome-targeted interventions offer an exciting therapeutic frontier, their clinical efficacy as a definitive method for preventing or treating human breast cancer remains unproven and should be viewed through an observational lens.

Beyond direct disease prevention, the microbiome profoundly modulates established cancer therapies, including chemotherapy, radiation, and immunotherapy [57,87,88]. In the context of chemotherapy, dysbiosis can induce the production of specific bacterial enzymes that can actively metabolize chemotherapeutic agents, thereby potentially altering drug toxicity and efficacy [89,90]. The administration of probiotics during chemotherapy has demonstrated potential clinical utility in maintaining gastrointestinal microbial balance and may help reduce treatment-associated toxicities, such as mucositis and diarrhea. Furthermore, emerging evidence suggests that specific microbial signatures may influence antitumor immune responses. These observations suggest that targeted microbiome manipulation, via fecal microbiota transplantation or precise probiotic administration, could hypothetically reverse immunotherapeutic resistance and improve overall patient survival outcomes by reconditioning the immunological milieu within the tumor microenvironment [73].

## 11. Emerging Research and Future Directions

### 11.1. Methodological Challenges and Multiomics Integration

Studying the breast microbiome presents a profound methodological challenge due to its ultralow microbial biomass. Thus, sequence data can be heavily confounded by contaminating DNA from laboratory reagents and extraction kits (often referred to as the “kitome”). Therefore, rigorous inclusion of blank experimental controls and advanced bioinformatic decontamination pipelines is mandatory in modern studies to distinguish authentic tissue-resident microbes from environmental contaminants. Furthermore, because DNA-based metagenomics cannot distinguish live bacteria from extracellular DNA fragments, integrating metatranscriptomics (RNA sequencing) is increasingly vital for confirming the presence of transcriptionally active, viable microbial communities within breast tissue.

**Figure 3:**
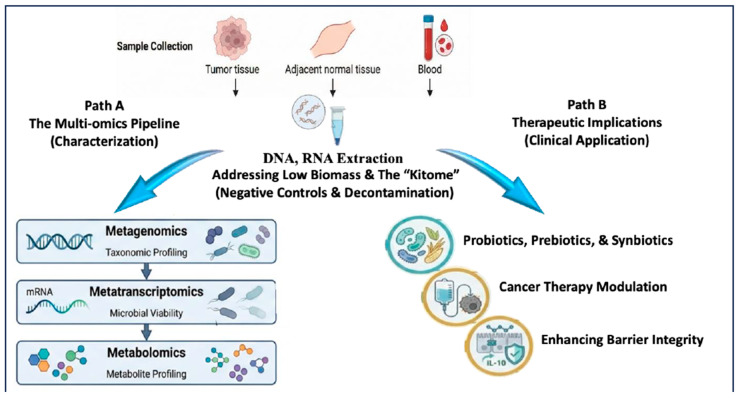
Schematic illustration of the comprehensive pipeline from clinical sample acquisition to multi-omics data integration and downstream therapeutic application. To mitigate the profound methodological challenges posed by the ultralow microbial biomass in breast tissue, the workflow mandates the integration of rigorous negative controls and advanced bioinformatic decontamination algorithms to systematically subtract environmental and reagent-derived (“kitome”) contaminants. Purified nucleic acids and metabolites are subsequently analyzed using a robust multiomics approach: DNA-based metagenomic sequencing for high-resolution taxonomic profiling, metatranscriptomics (RNA-seq) to validate viable, transcriptionally active microbial communities, and metabolomics to capture functional biochemical outputs. The translational trajectory highlights the application of these integrated data toward precision medicine, encompassing the deployment of targeted microbiome-modulating agents (probiotics, prebiotics, and synbiotics) and the strategic manipulation of the breast microbiota to potentiate the efficacy and attenuate the toxicity of conventional oncological regimens.

Beyond genomics, metabolic profiling is critical for identifying specific active molecular metabolic pathways within the breast microenvironment. Tang et al. [91] highlighted the potential of specific metabolites produced by beneficial microbiota in mitigating cancer development, as these compounds can modulate inflammation and carcinogenesis (Figure 3). Consequently, Ursell et al. [61] suggest that the discovery of specific microbial markers and metabolites linked to breast cancer may drive the development of innovative diagnostic instruments or targeted treatments intended to alter the microbiome to enhance patient outcomes. Table 2 summarizes and compares some methodologies used in breast microbiome research.

### 11.2. Precision Medicine and Microbiome-Based Prognostics

The main objective of personalized medicine is to customize treatment strategies to every individual based on unique properties; one potential area for this approach is the breast microbiome. Individual differences exist in the makeup of the breast microbiome, which can be attributed to environmental, genetic, and lifestyle factors. It is plausible that profiling the particular microbial composition of a patient’s breast tissue may identify distinct microbial signatures linked to therapy response and disease risk. For example, some species of bacteria have been associated with a higher risk of breast cancer, while other species could be preventive. To improve patient outcomes, personalized therapies may entail altering the microbiome to increase its beneficial components or decrease its detrimental ones [44]. One important strategy to design customized treatment involves analyzing the breast microbiome through metagenomic sequencing. Using this technology, microbial communities found in breast tissue may be thoroughly analyzed in order to determine the bacterial species and the genes that control their functions. With this knowledge, medical professionals may develop customized treatments that alter the microbiota to prevent or cure breast diseases. For instance, it could be advised to consume probiotics, prebiotics, or make particular dietary adjustments to encourage the proliferation of probiotic organisms and suppress the growth of disease-causing organisms [95]. Metabolomics and metagenomics can offer important insights into the functional status of the breast microbiome. The metabolites generated by microbial activity are essential for controlling cellular metabolism, inflammation, and immune function. Careful analysis of the metabolic profile in a patient’s breast tissue can provide insight into how the microbiome affects breast health. These data encourage the development of tailored therapy strategies that target certain metabolic pathways, which can enhance the effectiveness of current therapies. For instance, the discovery of metabolites with anti-inflammatory properties may result in the development of novel targeted therapy that reduces chronic inflammation, a recognized risk factor for breast cancer [93]. Nonetheless, the analysis and use of microbiome data in a therapeutic environment is on the rise and becoming more reliable due to developments in bioinformatics and machine learning [96]. The utilization of breast microbiome-based personalized medicine has significant potential to enhance disease prevention, diagnosis, and therapy. With further study in this area, more accurate and efficient therapies can be customized to the distinct microbial makeup of every patient’s breast tissue [97].

### 11.3. Ethical Considerations in Microbiome-Based Clinical Translation

Translating the findings of breast microbiome research to clinical practice presents a number of ethical issues and difficulties. Key concerns include patient autonomy and consent about the use, storage, and sharing of their microbiome data. This entails maintaining openness on the advantages and disadvantages, as well as privacy issues. Due to the complexity of microbiome research, maintaining patient autonomy is difficult but necessary, while ensuring informed consent by communicating this information clearly [98]. The possibility of unforeseen repercussions of microbiome-based therapies presents another ethical dilemma. For example, probiotics and other treatments that alter the microbiome may cause unanticipated health problems or dysbiosis. Thorough clinical studies are required to guarantee the effectiveness and safety of these therapies. Furthermore, a thorough evaluation of ethical principles is essential to safeguard participants from damage and guarantee that the benefits outweigh the risks [99]. Ethical challenges also arise regarding equity and access to microbiome-based medicines. These cutting-edge therapies could be costly and initially restricted to specialized facilities, which might exacerbate health inequities [100]. Fair and equal access to these developments must be guaranteed, taking insurance coverage into account and incorporating new therapies into public health initiatives. Ethical frameworks must be constructed to ensure equitable distribution and prevent the creation of a healthcare divide [101].

Finally, much remains unknown about the long-term effects of modifying the breast microbiota, which require follow-up of microbiome-based therapeutics over time and monitoring of the ongoing study. This entails being aware of the wider ecological influences on the microbiome, as well as any potential cross-generational consequences. In addition to immediate health outcomes, ethical research techniques should prioritize patients’ and communities’ long-term well-being.

### 11.4. Correlation Versus Causation: An Outstanding Question

A critical limitation in the current landscape of breast microbiome research is the heavy reliance on cross-sectional, observational study designs, which fundamentally restrict the ability to distinguish between correlation and causation [73]. While advanced metagenomic sequencing has successfully identified distinct microbial signatures associated with malignant breast tissue, the causal relationship remains ambiguous: whether microbial dysbiosis actively promotes carcinogenesis or if the altered tumor microenvironment acts as a catalyst that secondarily induces dysbiosis remains unclear. For example, enrichment of specific anaerobic or facultative bacteria within tumors could merely be a consequence of the tumor’s hypoxic and immunosuppressive architecture, which favors microbial colonization, rather than the microbes acting as the primary oncogenic trigger [72]. Furthermore, the ultralow biomass of breast tissue makes these studies particularly vulnerable to methodological artifacts. Challenges such as DNA contamination from laboratory reagents (kitome), environmental sources, and inter-study variability in DNA extraction protocols can skew correlative findings and artificially impose categorical structures on the data. To move beyond mere associations, the field urgently requires prospective, longitudinal human cohort studies that track microbiome dynamics over time [72].

## 12. Conclusions

The breast microbiome is important in preserving breast health, and imbalances can contribute to diseases, including breast cancer. Beneficial colonizing organisms, especially *Lactobacillus* and *Bifidobacterium,* enhance the production of anti-inflammatory cytokines and preserve the integrity of the epithelial barrier, thereby improving immune function and preventing chronic inflammation, which is associated with an increased risk of cancer [39]. Thompson et al. [38] established a link between breast cancer and dysbiosis, as evidenced by increased levels of pro-inflammatory bacteria, including *Staphylococcus* and Enterobacteriaceae, in tumor tissues. Furthermore, certain bacterial byproducts, such as SCFAs, inhibit breast tumor development [44]. Other studies also indicate the higher prevalence of certain bacteria in breast cancer tissues, such as *Methylobacterium* and *Ralstonia*, influencing tumor development [10]. The intricate character of the bacterial population in breast health and illness is highlighted by its interactions with host genetics, immunologic responses, and exogenous variables such as antibiotic use, lifestyle, and diet [11]. Further investigations of the breast microbiome are needed to fully understand how these microbial communities affect breast health and disease and evaluate the potential of therapeutic approaches. Furthermore, understanding how lifestyle factors, such as nutrition, hormone use, and antibiotic use, affect the breast microbiome can inform the implementation of tailored therapies to promote a balanced microbial community. A deeper understanding of the complex interactions among an individual’s unique microbiome composition, metabolic pathways, and the breast tissue microenvironment would yield valuable insights for developing precision therapeutics tailored to molecular pathways that drive breast tissue malignancy [102,103]. By monitoring microbial population shifts in response to a range of variables, including food, antibiotic use, aging, and hormonal changes, these studies could identify targets for prevention and indicators for early diagnosis. Longitudinal data can also help guide the development of personalized treatment tailored to an individual’s microbiome and unique personal history [11,38,39]. Further investigations employing cutting-edge methodologies, such as metagenomics and metabolomics, will enhance our understanding of the role of the breast microbiome in health and cancer. To ensure patient safety and fair access to new treatments, ethical issues in translating clinical research into therapeutic applications must be addressed. This extensive review underscores the importance of the breast microbiome, paving the way for subsequent studies to capitalize on its potential therapeutic benefits.

Despite growing evidence linking the breast microbiome to cancer development and progression, several fundamental questions remain unresolved. Whether microbial dysbiosis is a cause or a consequence of tumorigenesis remains unclear. The specific mechanisms by which microbes influence tumor initiation, immune modulation, and treatment response should also be investigated. Future research should focus on longitudinal and multiomics studies integrating metagenomics, transcriptomics, metabolomics, and spatial microbiome analyses to clarify causal relationships. Furthermore, standardized sampling protocols and contamination-control strategies are essential for improving reproducibility across studies. Research exploring host–microbe communication within the breast microenvironment may open new avenues for microbiome-based diagnostics, prognostic biomarkers, and targeted therapeutic interventions, including probiotics, microbiome modulation, and personalized treatment strategies.

## Figures and Tables

**Table 2:** Studies investigating the breast microbiome in breast cancer.

Method	Description	Strengths	Limitations	References
16S rRNA gene sequencing	Amplifies hypervariable regions of the bacterial 16S rRNA gene to identify taxa	Cost-effective; widely used; suitable for low-biomass samples	Limited taxonomic resolution; contamination sensitivity	[10,39,44]
Shotgun metagenomic sequencing	Sequences the total microbial DNA in tissue	Species/strain-level resolution; functional profiling	Expensive; computationally intensive	[92]
Metatranscriptomics	Profiles active microbial gene expression	Identifies metabolically active microbes	RNA instability; high cost	[44]
Metabolomics	Identifies metabolites produced by tumor-associated microbiota	Functional insight; host–microbe interaction data	Requires integrative interpretation	[93]
qPCR/targeted PCR	Quantifies specific bacterial taxa or genes	Highly sensitive; quantitative	Limited to known targets	[9]
Culture-based assays	Isolation of viable bacteria	Confirms viability; enables functional testing	Cannot detect unculturable taxa	[39]
Spatial transcriptomics/spatial microbiome mapping	Maps microbial signals within the tumor microenvironment	Reveals spatial organization; tumor–microbe interaction	Very costly; emerging field	[94]

## Data Availability

No new data were created or analyzed in this study. Data sharing is not applicable to this article.
